# Investigating the link between *APCI1307K* mutation and breast cancer in a Jordanian Arab population

**DOI:** 10.3389/fonc.2025.1557341

**Published:** 2025-06-27

**Authors:** Baha Sharaf, Hira Bani Hani, Anas Zayed, Maha Barbar, Suhaib Khater, Ahmad Hushki, Rashid Abdel-Razeq, Mohammad Titi, Reem Al-Halalsheh, Suleiman Mahafdah, Lin Ashour, Hikmat Abdel-Razeq

**Affiliations:** ^1^ Department of Internal Medicine, King Hussein Cancer Center, Amman, Jordan; ^2^ Department of Surgery, Royal Jordanian Medical Services, Amman, Jordan; ^3^ School of Medicine, the University of Jordan, Amman, Jordan

**Keywords:** APCI1307K, breast cancer, colon cancer, APC, germline genetic testing, hereditary cancer

## Abstract

**Introduction:**

*APCI1307K* missense mutation, welldescribed in Ashkenazi Jewish, is commonly encountered among Jordanian patients with solid tumors. In this study, we investigated the potential association between the *APC* gene (*I1307K* variant) and the risk of breast cancer among Jordanian Arab patients.

**Methods:**

All newly diagnosed patients with solid tumors were offered participation in a universal germline genetic screening study utilizing an investigational 84-gene panel. Patients were categorized based on whether they met or did not meet the criteria outlined in the National Comprehensive Cancer Network (NCCN) for genetic testing.

**Results:**

Among the screened cancer patients (*n* = 3,319), 136 (4.1%) had APCI1307K. Breast cancer was the most common primary tumor (*n* = 56, 41.2%). Among them, 41 (73.2%) had a screening colonoscopy, and 12 (29.3%) were found to have colorectal polyps, while 41.7% (5/12) had low-grade dysplasia. Of the 34 (25.0%) patients diagnosed with colorectal cancer, 23 (67.6%) exhibited tumors presenting as polyps, had concomitant polyps, or displayed background abnormalities with a polypoid nature.

**Discussion:**

These findings suggest that Arab individuals with the *APCI1307K* missense mutation are at higher risk of breast and familial colorectal cancers. The *APCI1307K* missense variant holds promise in informing screening and cancer prevention strategies. However, additional confirmation by larger studies is needed.

## 1 Introduction

Genetic mutations play a pivotal role in the initiation and progression of various cancers. The quest to understand the genetic basis of cancer susceptibility has led researchers to strive to understand the significance of specific genetic mutations in influencing an individual’s risk of developing malignancies. Among the numerous genetic variations associated with increased risks of certain conditions, the adenomatous polyposis coli (*APC*) gene, specifically the *I1307K* mutation, emerges as a genetic variant with notable implications for cancer predisposition (1, 2).

The *APC* gene is located on chromosome 5q21. It is an important tumor suppressor gene that encodes a multifunctional protein crucial for the maintenance of cellular homeostasis, and it regulates the Wnt/CTNNB1 (β-catenin) protein signaling pathway, cell adhesion, and cell cycle progression. Mutations in the *APC* gene have been linked to an elevated risk of colorectal cancer (CRC), as well as other cancers such as gastric and pancreatic malignancies (3–5).

The increased risk allele, the *I1307K* mutation, has emerged as a genetic variant with notable implications for cancer predisposition (2). It was identified in 1997 by Laken et al. as a missense variant mutation in codon 1307. It is formed by a transversion of thymine-to-adenine at nucleotide 1307, which leads to sequence changes from A3TA4 to A8. This results in the substitution of an isoleucine for a lysine, leading to a genetically unstable hypermutable region of the DNA, which increases the predisposition of somatic mutations (1, 6). *In vivo* and *in vitro* studies have confirmed that this variant predisposition causes slippage of the polymerase during DNA replication, causing an elevated risk of somatic truncating mutations occurring on this allele (1, 7, 8). Regardless of the experimental evidence, there is an inadequate association of the variant with Mendelian syndrome, which prevents its classification according to the consensus recommendations of the American College of Medical Genetics and Genomics and the Association for Molecular Pathology (9). There are conflicting interpretations with regard to the pathogenicity of the *APCI1307K* variant, as reported by multiple clinical laboratories and genomic databases, including ClinVar (10). While some classify it as a low-penetrance risk allele, others consider it a variant of uncertain significance (VUS) due to its modest cancer risk and its lack of strong association with Mendelian disorders. According to the Genome Aggregation Database (gnomAD v2.1.1), the *APCI1307K* variant (rs1801155) has an overall minor allele frequency (MAF) of 0.18% in the general population. However, it is significantly more prevalent in individuals of Ashkenazi Jewish descent, with a reported MAF of 3.6%, corresponding to approximately 1 in 28 individuals being a carrier (11).

The *APCI1307K* mutation has been linked to an increased susceptibility to cancer, prominently to CRC, mostly in the Ashkenazi Jewish population. A study published by Liang et al. showed that *APCI1307K* has a higher prevalence among Ashkenazi Jews (11.8%) than in non-Ashkenazi Jews (2.9%) and the non-Jewish population (0.92%) (12). Moreover, several studies have confirmed that the *APCI1307K* mutation has increased the risk of CRC in Ashkenazi Jews (~6%–10%). It is also present in ~2.5% of Sephardic Jews and in non-Jewish populations. It only represents less than 0.15% of the European, Asian, Latin, or African population (1, 2, 13, 14). On the other hand, the association of the *APCI1307K* mutation with increasing the risk of adenoma formation or transformation from a benign to a malignant polyp is still unclear. However, Houlston et al. reported that the *APCI1307K* mutation has a lower penetrance compared with familial adenomatous polyposis (FAP) and that the protein remains functional, adding a layer of complexity to our understanding of the genetic underpinnings of this prevalent malignancy (15). Nevertheless, based on the reported association of the *APCI1307K* mutation with the increased risk of CRC, the current National Comprehensive Cancer Network (NCCN) guidelines recommend CRC surveillance in individuals with the *APCI1307K* variant, regardless of ethnicity (16).

While CRC is a prominent manifestation, the impact of the *APCI1307K* mutation extends beyond the colorectal tissue, and there have been various reports on the potential risks attributable to this mutation and extra-colonic tumors, such as breast, prostate, ovarian, and skin, with debatable results (11, 17–21). Moreover, a strong correlation between *APCI1307K* mutation carriers and increased risk of pancreatic and lung cancers has also been reported (11). More interestingly, Woodage et al. concluded that *APCI1307K* mutation carriers are at risk of having any cancer (except non-melanoma skin cancer) compared with non-carriers, with an odds ratio (OR) of 1.5 (19).

As mentioned previously, several studies have addressed the role of *APCI1307K* in non-CRC. Some studies suggest a modestly increased risk of breast cancer in individuals carrying the *APCI1307K* mutation (8, 18, 22–24). Referring to the Wnt protein signaling pathway in breast cancer as similar to its role in CRC, the *APCI1307K* mutation influences the Wnt signaling pathway, causing dysregulation of its pathway, which can impact cell proliferation and differentiation, potentially contributing to the development of breast cancer. According to Valle et al., the prevalence of the *APCI1307K* mutation in non-Ashkenazi Jews with breast cancer was significantly higher compared with that in healthy individuals (OR = 1.73, 95%CI = 1.18–2.65, *p* < 0.01). On the other hand, in Ashkenazi Jews with breast cancer, there was no significant difference compared with healthy controls (11).

The association between *APCI1307K* mutation and CRC has been extensively studied with a particular focus on the Ashkenazi Jewish population. However, the association of the *APCI1307K* mutation with extra-colonic malignancies among non-Jewish has not been well studied. Therefore, this study aimed to explore the *APCI1307K* mutation and examine its implications in the development of cancer, focusing on breast cancer among the Jordanian population. By shedding light on this genetic alteration, we hope to deepen our understanding of its implications in order to emphasize the paramount importance of early detection, risk assessment, and potential therapeutic interventions.

## 2 Methods

### 2.1 Study participants

**Figure 1:**
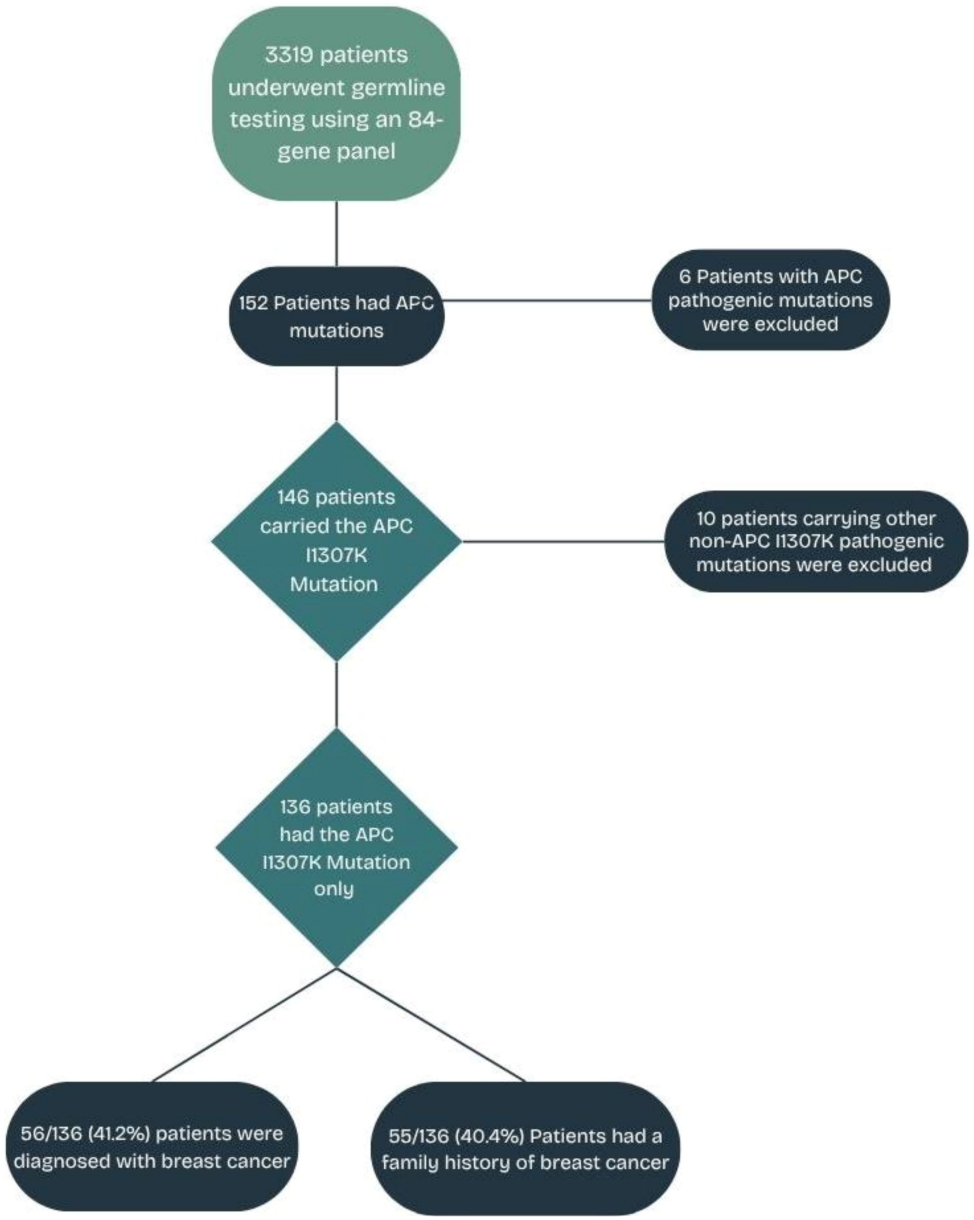
*APC I1307K* mutation study flowchart.

All newly diagnosed patients with solid tumors between March 2021 and December 2022 at King Hussein Cancer Center (KHCC) were invited to participate in a universal germline genetic screening study utilizing an investigational 84-gene panel testing. All patients were 18 years or older and were unselected for primary cancer type, stage of disease, age at diagnosis, or personal or family history of cancer. The Institutional Review Board (IRB) approved the study at KHCC (protocol no. 21-KHCC-27). All enrolled patients provided written informed consent. A retrospective analysis was conducted on all patients within the study group who are carriers of the *APCI1307K* missense mutation, irrespective of cancer type. To ensure that the study findings and the subsequent interpretations are centered specifically on the effects of the *APCI1307K* variant, patients with other pathogenic/likely pathogenic (P/LP) mutations (*n* = 10) were excluded from the analysis, as illustrated in **Figure 1**. Patients were categorized into two groups: 70 (51.5%) patients who met the criteria (in-criteria, IC) and 66 (48.5%) patients who did not meet the criteria (out-of-criteria, OOC) outlined in the NCCN guidelines v.1.2020 (25).

### 2.2 Genetic testing

All patients were evaluated during their first visit to KHCC by a medical, surgical, or radiation oncologist, where the nature of the study and an overview of the germline genetic testing (GGT) were discussed. If requested, patients were referred to a specialized genetic counseling clinic for additional counseling, consenting, and blood draw. The GGT cost was covered by insurance for patients who met the GGT criteria and by a competitive KHCC intramural research grant for those who did not meet the criteria. All GGT results were reviewed by genetic counselors and were disclosed to the patient by their oncologist. Patients with P/LP germline variants (PGVs) were offered post-testing genetic counseling. Whole-gene sequencing, DNA analysis, and variant interpretation were performed at Invitae Corporation, San Francisco, CA, USA, and the methods have been previously described (26, 27).

### 2.3 Data collection

The demographics, medical and family history, and the GGT results were extracted from electronic medical records and our institutional cancer registry. All data were de-identified, except for the study investigators.

### 2.4 Cascade testing

Cascade testing for at-risk family members was offered at almost no cost, if done within a period of 150 days of the patient’s finalized test result report. The number of cascade testing and the GGT results for patients with the *APCI1307K* missense mutation were retrospectively collected for this study.

### 2.5 Statistical analysis

Descriptive statistics were applied when appropriate to report the mean, median, standard deviations, and proportions. Statistical analyses were performed using STATA version 18. Differences in proportions were determined using Fisher’s exact test, with *p*-value <0.05 indicating a statistically significant difference.

## 3 Results

### 3.1 Participant characteristics

Overall, 3,319 patients with solid tumors underwent genetic testing as part of a universal genetic study (28). A total of 460 PGVs were detected in 428 (12.9%) patients. There were 146 patients who tested positive for the increased risk allele *p.I1307K* in *APC* (representing 34.1% of the patients with positive findings or 4.4% of the overall cohort) (28). There were 10 (6.8%) patients who had P/LP variants in genes other than *APCI1307K* (a list of the genes and mutations is provided in **Supplementary Table S1**). A total of 136 (93.2%) patients had only the *APCI1307K* mutation and will be the focus of this analysis. The median (range) age at cancer diagnosis was 52 years (19–80 years), and 84 (61.8%) were women (**Table 1**).

**Table 1:** Participant Characteristics (n=136).

Characteristics		Number of patients with *APC* I1307K	(%)
Age (years	≤45*	49	36.0
	> 45	87	63.9
Sex	Female	84	61.8
	Male	52	38.2
Primary cancer diagnosis	Breast	56	41.2
	Colorectal	34	25
	Lung	7	5.1
	Renal/bladder	7	5.1
	Esophageal/Head and Neck	5	3.7
	Pancreatic	4	2.9
	Ovarian	4	2.9
	Endometrial/uterine	4	2.9
	Gastric	4	2.9
	Sarcoma	4	2.9
	Testicular	3	2.2
	Prostate	2	1.5
	Cervical	1	0.7
	Brain	1	0.7
Disease stage	Metastatic	48	35.3
	Early	88	64.7
Family history of cancer	Yes	112	82.4
	No	24	17.6
NCCN GGT criteria status	In criteria (IC)	70	51.5
	Out of criteria (OOC)	66	48.5

* Age 45 was used as per the NCCN guidelines (2020) as a cut off age for germline genetic testing for breast cancer. [29] NCCN: National Comprehensive Cancer Network GGT: Germline genetic testing.

### 3.2 Primary cancers

**Figure 2:**
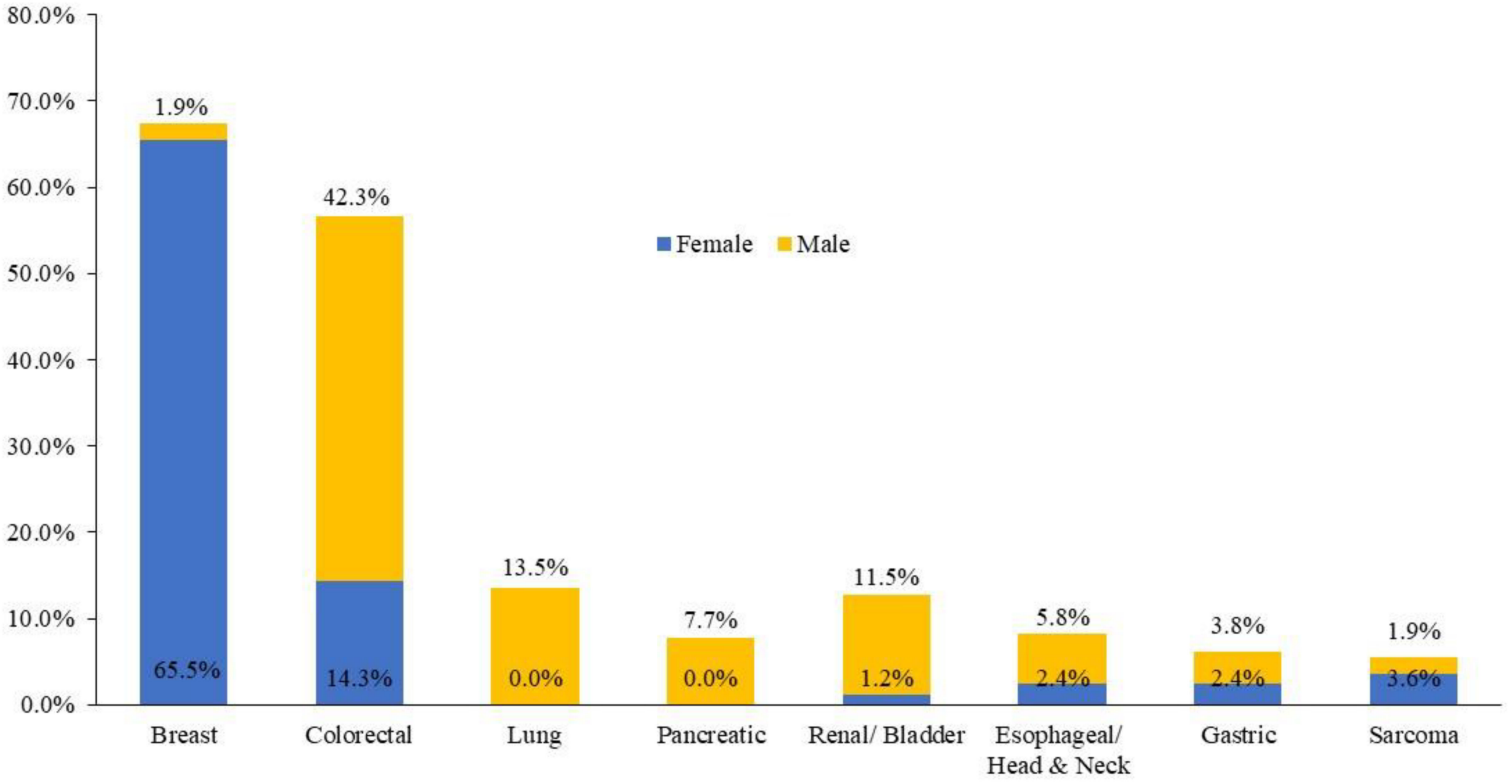
Distribution of the cancer types within each gender subgroup among *APC I1307K* carriers.

Among the 136 patients with the *APCI1307K* mutation, breast cancer was the most common primary tumor, identified in 56 (41.2%) patients, followed by CRC (*n* = 34, 25%), lung cancer (*n* = 7, 5.1%), and renal/bladder cancer (*n* = 7, 5.1%). The distribution of cancer by site and gender is illustrated in **Figure 2**. Furthermore, 14 (8.3%) patients harboring the *APCI1307K* mutation exhibited a history of antecedent malignancies; 5 (35.7%) patients had breast cancer as a second neoplasm.

Among the 56 patients diagnosed with breast cancer, 54 had invasive breast cancer and two had ductal carcinoma *in situ* (DCIS). The receptor status was available for 48 patients. Of these, 13 (27%) patients were HER2-positive, while 2 (4%) had triple-negative breast cancer. A total of 31 (65%) patients had tumors that were estrogen receptor (ER)- and/or progesterone receptor (PR)-positive and HER2-negative, which aligns with the most common breast cancer subtype globally.

Among the DCIS cases, one was ER- and PR-positive, while another was negative for both receptors. In addition, one patient had two distinct primary breast cancers with different receptor profiles: one tumor was ER/PR-positive and HER2-negative, while the other was ER/PR-positive and HER2-positive. The receptor status was unknown in 6 (11%) cases.

During follow-up, 3 (5.4%) of the 56 enrolled patients with breast cancer developed contralateral breast cancer. Screening colonoscopy was performed in 41 (73.2%) patients with breast cancer, which found 12 (29.3%) patients to have polyps (range, 1–3) and 5 (41.7%) with low-grade dysplasia. Colonoscopy was not performed in the other 25 patients with breast cancer due to patient refusal, the advanced stage of their primary cancer, and not meeting the age criteria for screening.

On the other hand, among the 34 patients with CRC, 23 (67.6%) had tumors presenting as polyps or exhibited concomitant polyps (range, 1–3) or displayed abnormalities in the background that were polypoid in nature. A noteworthy finding revealed that 7 (20.6%) patients with CRC had a positive family history of breast cancer.

### 3.3 Family history of malignances

**Figure 3:**
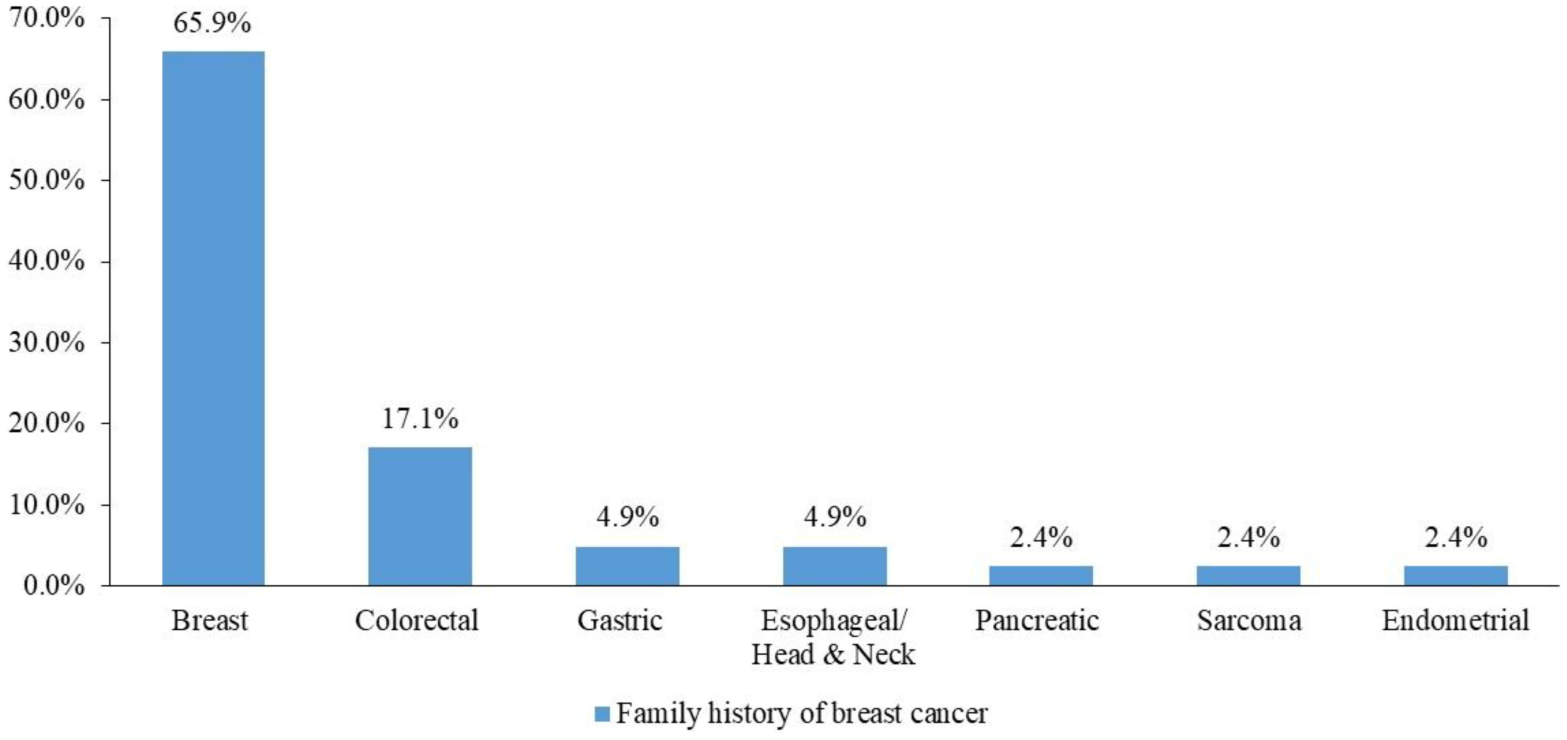
Family history of breast cancer among cancer types.

In total, 108 patients had a positive family history of malignancies, and 41 (37.9%) had a family history of breast cancer (**Figure 3**).

### 3.4 Analysis of other pathogenic/likely pathogenic mutations

To ensure the accuracy and precision of the results, 10 (6.8%) patients were excluded from the analysis due to having P/LP variants in genes other than *APCI1307K*. A comprehensive list of the excluded genes and mutations can be found in **Supplementary Table S1**. Implementing this exclusion criterion strengthened the validity and reliability of the results, allowing for a more focused exploration of the *APCI1307K* variant within the scope of the study. This approach enhanced the integrity of our research and provided a clearer understanding of the targeted genetic factors under investigation.

Notably, only three patients with the *APCI1307K* variant had additional variants in the *APC* gene, which were classified as either benign or a VUS. Although the number was small, these co-occurring variants may suggest a possible genetic pattern that could be important and therefore warrant further exploration. These additional heterozygous missense variants were reviewed. The first variant, c.1276G>A (p.Ala426Thr) in exon 10, is classified as a VUS based on multiple ClinVar submissions and Franklin by Genoox, with limited evidence supporting pathogenicity. The second variant, c.4349G>A (p.Arg1450Gln) in exon 16, is also a missense VUS, although it has been reported in some cases of FAP, suggesting a potential low-penetrance role. The third variant, c.3347G>A (p.Gly1116Asp), also located in exon 16, is similarly classified as a VUS, with no definitive clinical significance established. The presence of these additional variants alongside *APCI1307K* raises the possibility of a cumulative or modifying effect on cancer risk, although current evidence does not confirm a pathogenic interaction. Further functional studies and segregation analysis are needed to clarify any clinical relevance of this combination.

### 3.5 Cascade testing

**Figure 4:**
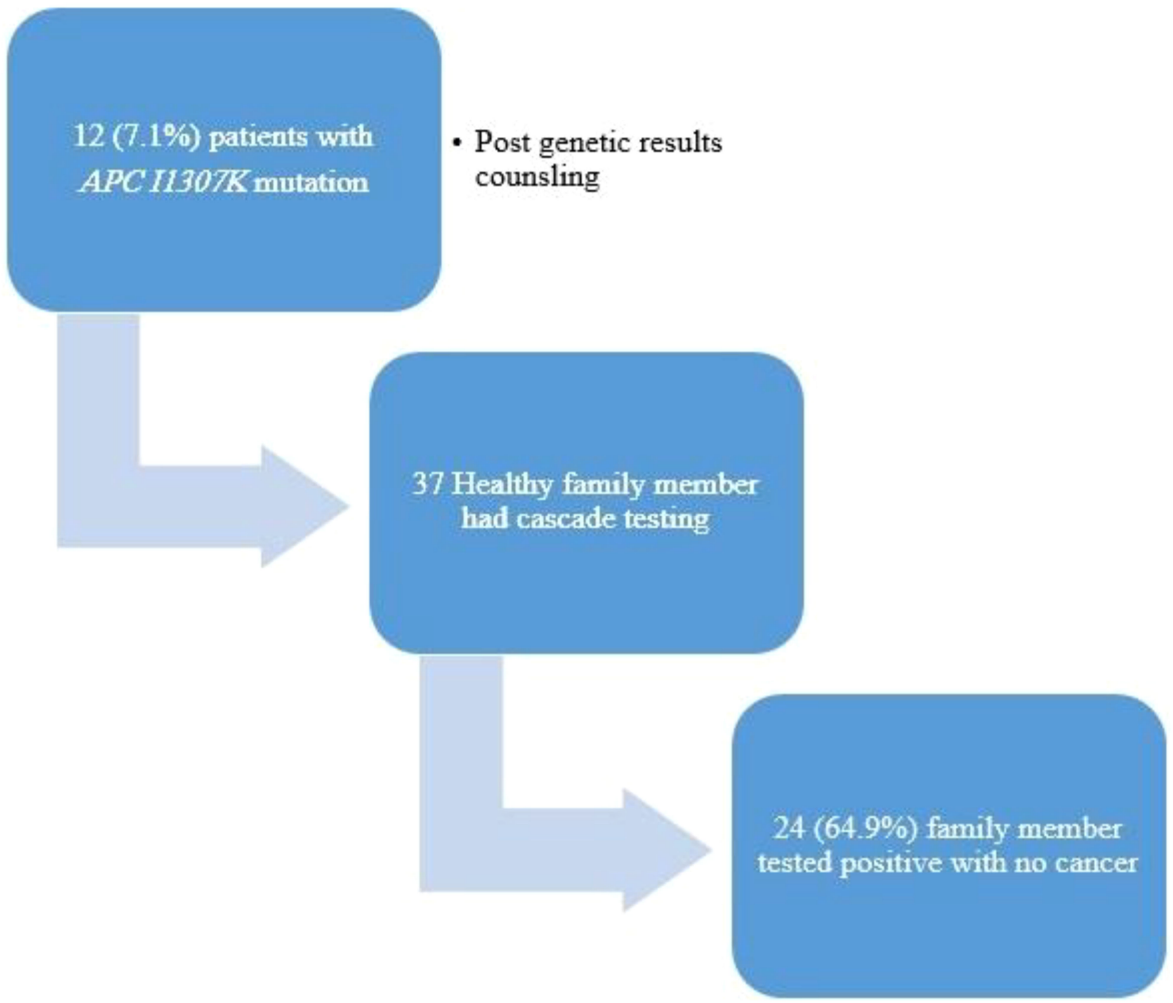
Cascade testing uptake and outcomes.

The family members (*n* = 37) of 12 patients (7.1%) underwent cascade testing, with 24 (64.9%) of them resulting positive for the *APCI1307K* mutation with no cancer diagnosis (**Figure 4**).

## 4 Discussion

The association of the *APCI1307K* variant with an elevated risk of CRC is well studied (1, 15, 20). However, the landscape becomes markedly nuanced when exploring its potential correlation with other cancer types in diverse ancestral backgrounds, especially beyond the Jewish population. Previous studies have shown that 6% of the Ashkenazi Jewish population are carriers of the *I1307K* mutation of the *APC* gene, and such prevalence is not well established in other ancestries, including Arab (11). This study is the first to investigate the pattern and frequency of the *APCI1307K* mutation in the Arab population and is the largest of its kind so far to correlate this mutation with breast cancer.

Multiple studies, however, have failed to link the *APCI1307K* mutation with extra-colonic tumors. The study by Woodage et al. showed no increased risk of other cancers beyond CRC among Ashkenazi Jewish carriers of the *APCI1307K* mutation, irrespective of their *BRCA* status (19). A parallel conclusion emerged from the study by Nathanson et al. on non-Ashkenazi Jews, reinforcing the complexity of the genetic factors at play (29). Furthermore, Poynter et al. failed to establish a clinically meaningful connection between *APCI1307K* and susceptibility to prostate cancer (23). Similarly, Abrahamson et al. found no heightened risk in patients with ovarian cancer carrying the *APCI1307K* mutation (30, 31). On the other hand, various studies have suggested a correlation between *APCI1307K* mutation and extra-intestinal tumors such as breast (32), lung (33), pancreatic (34), gastric (35), kidney (36), and skin cancers (37), but with lower rates than that in CRC. In terms of breast cancer, Redston et al. reported an OR of 1.5 among *APCI1307K* mutation carriers with breast cancer (21). In addition, Woodage et al. revealed a higher frequency of breast cancer among first-degree relatives who carry this variant (OR = 1.4, 95%CI = 1.1–1.8, *p* = 0.01) (19). This association was also established for other cancers. An extensive study that involved white, non-Ashkenazi Jews reported that carriers of the *APCI1307K* mutation had a significantly higher risk of several cancers, including colorectal (OR = 1.95, 95%CI = 1.39–2.73, *p* < 0.01), melanoma (OR = 2.54, 95%CI = 1.57–3.98, *p* < 0.01), breast (OR 1.73, 95%CI = 1.18–2.65, *p* < 0.01), and prostate cancer (OR = 2.42, 95%CI = 1.45–3.94, *p* < 0.01) (11). A high rate of the *APCI1307K* mutation in lung, ovarian, pancreatic, and renal cancer was also reported among the white, non-Ashkenazi Jewish population (11). Furthermore, the study by Minas et al. showed a correlation between *APCI1307K* mutation and an increased risk of a somatic genomic and/or an epigenomic landscape of prostate cancer (38). However, the rate of cancer predisposition for this extra-colonic cancer was much lower than that for the risk of CRC.

Moreover, it was reported that patients with the *APCI1307K* mutation have an increased risk of any cancer, but the OR in men was higher in lung (OR = 7.3, 95%CI = 2.58–20.7, *p* < 0.0001), pancreatic (OR = 3.71, 95%CI = 1.71–8.03, *p* < 0.001), urinary tract (OR = 4.5, 95%CI = 1.49–13.57, *p* < 0.001), and skin cancers (OR = 3.25, 95%CI = 1.44–7.36, *p* < 0.001) (11). On the other hand, women had a higher risk of breast (OR = 2.84 95%CI = 1.74–4.66, *p* < 0.0001) and skin cancers (OR = 4.81, 95%CI = 2.90–7.97, *p* < 0.0001) (11). However, in this study, breast, colorectal, lung, renal/bladder, esophageal/head and neck, pancreatic, ovarian, and endometrial cancers were the most encountered tumors in carriers of *APCI1307K*.

More interestingly, several European studies failed to show the *APCI1307K* mutation in a white, non-Ashkenazi Jewish population with colorectal cancer, including studies from England (*n* = 134) (17), Sweden (*n* = 194) (39), and Croatia (*n* = 73) (40). In addition, an African American, Italian, Finnish, and Hawaiian–Japanese study included 345 participants with no *APCI1307K* mutation carriers (2). However, in a Norway study, 1 out of 210 patients with CRC was found to be an *APCI1307K* mutation carrier, and the patients were reported to be Jewish (41), which confirms the findings of Liang et al. reporting a prevalence of only 0.92% (95%CI = 0.51–1.66) of *APCI1307K* among non-Jewish patients with CRC from nine different studies (12).

Interestingly, in a study on 120 Egyptian colorectal cancer patients compared to 100 healthy controls, the *APCI1307K* carrier frequency was significantly higher among patients with colorectal cancer compared with the controls (18.3% *vs*. 9.0%; OR = 2.58, 95%CI = 1.09–6.09, *p* = 0.03) (42).

In this study, the prevalence of the *APCI1307K* mutation among patients with different cancers was 4.4%, while the literature has extensively documented its occurrence in approximately 6% of the Ashkenazi Jewish population.

Our study is in agreement with all prior research and stands as a pioneering and exclusive investigation addressing the prevalence of the *APCI1307K* mutation among the Arab population in the Middle East. Moreover, 102 patients with extra-colonic cancers were identified to have *APCI1307K*, and 64 (62.7%) underwent screening colonoscopy. It might be concerning that 21 (32.8%) patients had one to three polyps, including low-grade (*n* = 11) and high-grade dysplasia (n = 1), and one patient had a tumor with a polypoid background.

In addition, it highlights the familial implications of the study findings. Among the patients with *APCI1307K*, 37 family members of 12 patients (7.1%) underwent cascade testing. Remarkably, of these tested family members, 24 (64.9%) individuals were found positive for the *APCI1307K* mutation despite not having cancer themselves. This finding underscores the importance of cascade testing in identifying individuals at higher risk due to familial genetic mutations, even in the absence of a cancer diagnosis.

In our institution, we follow the NCCN guidelines for *APCI1307K*, which recommends that patients with *APCI1307K* undergo a colonoscopy at age 40 years with a follow-up every 5 years (43). However, based on the results, breast cancer screening and early detection should be considered as well. Furthermore, the identification of many family members harboring the *APCI1307K* mutation highlights the importance of cascade testing of those family members who are at risk.

This study has some limitations. Firstly, our institution offered GGT for clinical purposes only. Although we treat over 50% of patients with cancer in the country, the included patients might not reflect national trends. Secondly, although all of the included patients are Arabs, Jordanians might not represent other Arab nationalities, especially North Africans. Thirdly, the finding of this *APC* variant in non-CRC patients was incidental when we started using an expanded multi-gene panel testing as part of a universal GGT study. Despite this potential bias, the population enrolled in our study is one of the most extensively tested. Finally, the rates presented in this study represent patients with cancer: we have not studied the prevalence of these variants in unaffected controls. Our research methodology examined the effect of the *APCI1307K* mutations in isolation from the impact of other known pathogenic mutations, such as *BRCA1* and *BRCA2*, and the results cannot be attributed to other known pathogenic variants.

## 5 Conclusions

Although *APCI1307K* is unexpectedly common in our cohort of Arab patients with cancer, in addition to CRC, the rates of this *APC* variant are also unexpectedly high among patients with breast cancer, which led to our recommendation of adding the *APC* gene to at least the multi-gene panel used in the routine GGT of patients with colorectal and breast cancers. Further studies are needed to verify these unexpected findings among Arab and other ethnic groups. In addition, additional research is required to define the clinical importance of the identification of this variant among patients and their at-risk unaffected family members.

## Data Availability

The datasets presented in this study can be found in online repositories. The names of the repository/repositories and accession number(s) can be found in the article/**Supplementary Material**.
