# Supplementary material for: Investigating the link between APCI1307K mutation and breast cancer in a Jordanian Arab population
Source: Front Oncol. 2025 Jun 27;15:1557341. doi: 10.3389/fonc.2025.1557341 (PMC12245844; doi:10.3389/fonc.2025.1557341)
Supplement: Supplementary file 1 [file Table1.docx]

Supplementary Material

# Supplementary Figures and Tables

**Table 1.** List of genes and pathogenic/likely pathogenic mutations

| Genes | Exon/ Intron | Nucleotide Changes | Amino Acid Change | Variant Type | Database Report | Frequency |
| --- | --- | --- | --- | --- | --- | --- |
| BRCA1 | Exon 15 | Deletion | Deletion | Deletion | Yes | 1 |
| BRCA2 | Exon 11 | c.2254_2257del | p.Asp752Phefs*19 | Deletion | Yes | 3 |
| BRCA2 | Exon 11 | c.3847_3848del | p.Val1283Lysfs*2 | Deletion | Yes | 1 |
| CHEK2 | Exon 4 | c.499G>A | p.Gly167Arg | Missense | Yes | 1 |
| MUTYH | Exon 3 | c.254A>G | p.His85Arg | Missense | Yes | 1 |
| NTHL1 | Exon 2) | c.268C>T | p.Gln90* | Nonsense | Yes | 1 |
| RET | Exon 15) | c.2671T>G | p.Ser891Ala | Missense | Yes | 1 |
| TP53 | Exon 8 | c.817C>T | (p.Arg273Cys) | Missense | Yes | 1 |

**Table S2.** Gender-Specific Distribution of Cancer Types Among APC I1307K Carriers

| Cancer Type/ Gender | Female (N (%)) | Male (N (%)) |
| --- | --- | --- |
| Total Number | 84 (61.8%) | 52 (38.2%) |
| Breast | 55 (65.5%) | 1 (1.9%) |
| Colorectal | 12 (14.3%) | 22 (42.3%) |
| Lung | 0 (0.0%) | 7 (13.5%) |
| Pancreatic | 0 (0.0%) | 4 (7.7%) |
| Renal/ Bladder | 1 (1.2%) | 6 (11.5%) |
| Esophageal/ Head & Neck | 2 (2.4%) | 3 (5.8%) |
| Gastric | 2 (2.4%) | 2 (3.8%) |
| Sarcoma | 3 (3.6%) | 1 (1.9%) |
